# Review of Allelopathy in Green Tides: The Case of *Ulva prolifera* in the South Yellow Sea

**DOI:** 10.3390/biology13060456

**Published:** 2024-06-20

**Authors:** Yinqing Zeng, Xinlan Yang, Zhangyi Xia, Runze Chen, Faqing He, Jianheng Zhang, Peimin He

**Affiliations:** 1 College of Oceanography and Ecological Science, Shanghai Ocean University, Shanghai 201306, China; zyq70442083@gmail.com (Y.Z.); y496898726@163.com (X.Y.); xzy19028@163.com (Z.X.); 18854318218@163.com (R.C.); fqhe0906@163.com (F.H.); 2 Key Laboratory of Exploration and Utilization of Aquatic Genetic Resources, Ministry of Education, Shanghai Ocean University, Shanghai 201306, China; 3 Co-Innovation Center of Jiangsu Marine Bio-Industry Technology, Jiangsu Ocean University, Lianyungang 222005, China

**Keywords:** green tide, allelopathy, allelochemicals, *Ulva prolifera*, physiological mechanism, marine monitoring

## Abstract

**Simple Summary:**

The spread of large green algae in oceans causes green tides, particularly in the South Yellow Sea of China, where *Ulva prolifera* has led to the world’s largest green tide events. This review looks at how allelopathy, a process where plants release chemicals to affect the growth and development of other plants, influences green tide dynamics. We focus on four main types of these allelochemicals—fatty acids, aldehydes, phenols, and terpenes—explaining how they influence the algae’s growth and behavior. We also discuss new methods for quickly detecting these allelochemicals and how these methods can help monitor green tides. By combining allelochemical detection with advanced technologies like satellite observations and environmental DNA analysis, we can better understand and manage green tides.

**Abstract:**

The proliferation of large green macroalgae in marine environments has led to the occurrence of green tides, particularly in the South Yellow Sea region of China, where *Ulva prolifera* has been identified as the primary species responsible for the world’s largest green tide events. Allelopathy among plants is a critical factor influencing the dynamics of green tides. This review synthesizes previous research on allelopathic interactions within green tides, categorizing four extensively studied allelochemicals: fatty acids, aldehydes, phenols, and terpenes. The mechanisms by which these compounds regulate the physiological processes of green tide algae are examined in depth. Additionally, recent advancements in the rapid detection of allelochemicals are summarized, and their potential applications in monitoring green tide events are discussed. The integration of advanced monitoring technologies, such as satellite observation and environmental DNA (eDNA) analysis, with allelopathic substance detection is also explored. This combined approach addresses gaps in understanding the dynamic processes of green tide formation and provides a more comprehensive insight into the mechanisms driving these phenomena. The findings and new perspectives presented in this review aim to offer valuable insights and inspiration for researchers and policymakers.

## 1. Introduction

A “green tide” is the result of green macroalgae, such as *Ulva prolifera*, growing and spreading quickly in the maritime environment under specific circumstances. Throughout the last fifty years, this phenomenon has been observed in numerous coastal regions of the world [1,2,3]. Although green tides have the potential to encourage the long-term storage of carbon by releasing huge volumes of dissolved organic carbon that is difficult to degrade into offshore waters, it upsets the balance of the marine ecosystem and burdens the economy [4]. Green tides may also directly worsen the quality of the environment by changing the composition of the phytoplankton community and maybe encouraging the proliferation of some microalgae, including *Aureococcus anophagefferens*, which can then increase brown tides [5]. Increasingly common, these biological occurrences have a significant ecological and economic influence on coastal regions [6,7,8].

**Figure 1:**
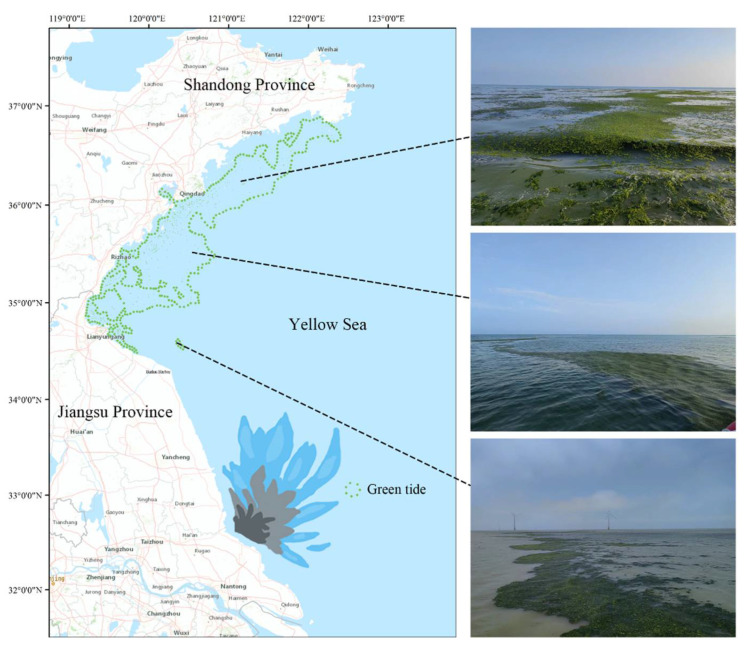
Green tide of *U. prolifera* in the South Yellow Sea in 2022. From May to July each year, large-scale green tides move northwards from Jiangsu to the Shandong Peninsula. Satellite images cited from previous work [17].

Since 2007, the Yellow Sea region of China has experienced regular instances of green tides dominated by *U. prolifera* [3]. The origin, breakout, and migration routes of the South Yellow Sea green tide have been thoroughly studied by scientists throughout the last ten years [9,10,11]. Utilizing a combination of remote sensing monitoring and on-site investigations, Liu et al. (2009) proposed that the *Neopyropia* aquaculture areas in northern Jiangsu might be the primary source of the SYS green tide [9], with this viewpoint being widely accepted in subsequent studies [12,13]. The vast rafts dispersed in the northern Jiangsu *Neopyropia* aquaculture regions offer plenty of attachment substrates for the development of huge amounts of green macroalgae [12]. Every year between April and May, green macroalgae affixed to the rafts separate during the retrieval process, and as water temperatures rise, *U. prolifera* takes the front stage. Driven by wind and ocean currents, floating *U. prolifera* then travels northward, creating widespread green tide phenomena (Figure 1) [13]. Due to allelopathic interactions, *U. prolifera* affects the species composition in the marine environment and encourages its growth during green tide occurrences. Still, these allelopathic interactions are fairly little researched, and the present research on the processes behind the formation of green tides is rather incomplete [14,15,16]. Better knowledge of the allelopathic mechanisms during green tide occurrences and the provision of management strategies based on this knowledge will be essential to effectively manage and reduce the frequency of green tide episodes.

Known as allelochemicals, bioactive substances generated by secondary metabolic activities are essential to the communication and signal exchange between plants and the environments around them [18]. These substances can affect the development and reproduction of nearby species, therefore regulating biological interactions within the ecology. More precisely, allelochemicals give plants an edge over other plants in competition and shield them against pathogens and herbivores [19]. Allelochemicals comprise a wide range of intricate chemical structures and biological activities; some examples are alcohols, phenols, terpenoids, alkaloids, and chemicals containing halogen [19,20]. Of them, phenolic chemicals have drawn the attention of a great deal of research since they are essential for controlling plant growth and seed germination [21]. Significant herbicidal effects, inhibition of other plant development, and disruption of other plant physiological activities are all possible with terpenoids and alkaloids [22,23]. Plant root secretion, aqueous solution release, volatilization, and plant residue breakdown are just a few of the ways allelochemicals are produced. These pathways allow allelochemicals to efficiently act on neighboring biomes, thereby influencing their composition and function [24,25]. The release of this family of compounds gives invasive plants a competitive edge in their new surroundings. By limiting native plant development, disrupting microbial symbiosis, and changing the distribution of soil nutrients, these chemicals help invasive species spread and settle [26]. Then, allelochemicals have a wide range of environmental consequences. They demonstrate their essential and significant function in natural ecosystems by influencing not only the relationship between plants but also other ecological communities including soil microbes, mycorrhizal fungi, and invertebrates [27].

Allelopathy among aquatic plants is crucial to the marine ecological environment. The green tide process is greatly aided by these compounds, which may be stimulating or inhibiting, therefore controlling competition and biodiversity in the environment. Citing Teneva et al. (2023) and Chia et al. (2021), cyanobacteria, for instance, release two toxins, microcystin-LR and cylindrospermopsin, which prevent green macroalgae from growing [28,29]. Not only can microalgae interact chemically, but macrophytes and microalgae do too. Seagrasses inhibit the growth of toxic algae by allelopathy, hence preventing their spread [30]. Spent in the intertidal zone, invertebrates like sponges release particular compounds (such as β-sitosterol) to compete for nutrients and living space, which can also impact the physiological processes of other algae [31]. Additionally important is allelopathy among benthic microbes, which through the transmission of chemical signals, affects the composition and ecological efficacy of microbial communities [32]. Significant allelopathic inhibitory effects on *Spartina alterniflora* have been shown for extracts from *U. prolifera*, including valine, hexanedioic acid, and bis(2-ethylhexyl) ester [33]. In ecosystems, allelopathy has a strong and complicated function. It is significant to biodiversity preservation and interspecies competition. It shows how deeply intertwined and dependent ecosystem members are [34,35].

Enhanced by allelopathy, the persistence of green tides in the ocean presents complex ecological and economic problems [36]. Even if allelopathy in marine ecosystems should receive similar attention, particularly during green tide outbreaks, previous research has mostly concentrated on allelopathy in terrestrial settings [37]. This article tries to provide a comprehensive review of works on this topic to highlight the importance of allelopathy in managing species competition and maintaining biodiversity and ecological balance in maritime habitats. In Table 1, we present the key allelochemicals involved in the process of green tide formation. By closely analyzing the effects of allelopathy on the green tide, we hope to add to the future monitoring, prevention, and management plans for the marine environment, which are necessary to ensure its sustainability and health.

## 2. Allelochemicals and Allelopathy Mechanisms in the Green Tide Process

### 2.1. Fatty Acids

Because fatty acids greatly affect the development and health of both plants and microbes, it is essential to understand their function in ecosystems. To emphasize their significance even further, these substances also function as allelochemicals. Fatty acids play a crucial role in various biological processes, including the regulation of cell structure, the management of oxidative stress responses, and the modulation of gene expression. First of all, changes to the architecture of cells may result from fatty acids. Allelochemicals have been shown in earlier research to influence cell division, elongation, and membrane function [50,51]. For instance, a transmission electron microscopy investigation of the *Heterosigma akashiwo* study revealed that the addition of linoleic acid significantly altered the morphology of algal cells [52]. Furthermore, inside cells, oxidative stress, which is associated with reactive oxygen species (ROS) production, leads to increased lipid peroxidation and can be influenced by fatty acids. As stated by Pinto et al. (2013), this reaction is a typical physiological process [53]. In *H. akashiwo*, linoleic acid boosts the intracellular antioxidant ability to reduce damage [54,55]. Moreover, the way fatty acids regulate gene expression has a significant impact on the body’s defenses against metabolic changes and the onset of illnesses. The impacts listed are displayed by epigenetic pathways, as Gniazdowska et al. (2004) have shown [56].

Fatty acids are important participants in the chemical communication network of the intricate undersea ecosystems. As Teneva et al. (2023) have pointed out, important allelopathy between algae and phytoplankton [28]. To an expert on the subject, the significance of fatty acids like hexadecanoic acid and octadecenoic acid, which is from *Ulva linza*, can influence the growth of phytoplankton populations along the shore [39]. And, *U. prolifera*’s spore germination can be inhibited by nonanoic acid [40]. Changes in the local lighting conditions affected the increased production of these fatty acids [57]. Under high light, *U. prolifera* will change their carbon storage and improve their physiological photosynthetic activities. This strategy is mostly shown in the change from C3 to C4 photosynthesis [46]. The biological modification greatly enhances fatty acid synthesis and enables it to continue to be active in harsh conditions [46]. Furthermore, included in this survival strategy is precisely controlled energy expenditure and carbohydrate metabolism [58]. On the other hand, the activity of a triacylglycerol synthetase known as DGAT is increased in the cells of *U. prolifera* when faced with the difficulties of high salinity and temperature. This enzyme helps to accumulate fatty acids and is essential for *U. prolifera*’s ongoing survival [42]. Zhuo et al. (2022) descended further into the genome of *U. prolifera* and discovered that genes involved in fatty acid synthesis in *U. prolifera* became more active in the event of significant salinity fluctuations [59]. Stress tolerance genes are also intimately associated with fatty acid metabolic pathways. These show that fatty acids significantly influence stress and the control of biological responses and that these pathways are essential to the environmental adaptability and growth of algae [59]. Particularly acidifying circumstances significantly affect the levels of polyunsaturated fatty acids and palmitic acid [38].

### 2.2. Aldehydes

Aldehydes can have a significant impact on the growth and development of nearby plants [60]. These compounds can chemically interact with vital plant macromolecules like proteins and DNA, affecting the plant’s general physiology and survival capacity [50]. Aldehyde-related mostly harmful effects include genetic changes and DNA damage [45,61]. The chemical structures of biomolecules control the many aldehyde functions. Aldehydes having α and β-unsaturated carbonyl groups prefer to stick to more flexible biomolecules than others [62]. Targeted and upsetting specific physiological functions in plants, aldehydes can impact cellular activity, metabolism, and respiration. Shirgapure et al. (2020) conducted research demonstrating how environmental variables, including variations in temperature and soil quality, influence the allelopathic effects of aldehydes and other allelochemicals. These abiotic stress factors exert physiological pressure on plants through allelopathic interactions [63]. Plant relationships and adaptation could be changed by outside factors on the synthesis and secretion of allelochemicals [64]. As is well known, plants release volatile organic compounds like aldehydes that strengthen their defenses against diseases and insects. Additionally contributing to the structure and ecological activities of plant communities are these substances’ allelopathy [65,66]. Aldehydes are thus crucial to plant allelopathy as well as to the dynamics and overall well-being of plant communities. In specific environmental settings, they engage with biomolecules. This allelopathy emphasizes the significance of aldehydes in maintaining ecological balance and plant interactions [67].

Without aldehydes, green macroalgae cannot survive, and in marine environments, aldehydes also allow green macroalgae to communicate with other living things. Among the phytoplankton, green macroalgae and diatoms release these substances into the water that are necessary for the development of green tides [68]. The hazardous aldehydes 2-trans and 4-trans-decadienal aldehyde are among those generated by some diatoms in both freshwater and marine environments, these aldehydes also reduce the chances of diatom survival, initiating a self-regulating ecological process [41]. Through its regulation of growth rates and induction of cell death, this phenomenon indirectly influences the dynamics of the green tide [69,70]. Variations across species and environmental factors determine how allelopathy affects biological populations. Green macroalgae development has been inhibited by the aldehydes emitted by the red tide dinoflagellate *Karenia brevis*, as laboratory research has shown. It is noteworthy, though, that because of the complex biological diversity found in natural settings, this effect would be less noticeable [71]. Citral was also found to have inhibitory effects on the spore germination of *U. prolifera* [40]. Because secondary compounds produced by the algae might impede the formation of other biological communities, green tide algae accumulate in specific conditions [72].

### 2.3. Phenols

Mostly produced by the acetic acid and shikimic pathways, phenols have given plants a fundamental environmental stress line [73]. Particularly in the competition amongst species, these compounds have been crucial to the development and evolution of plants. For instance, invasive plants can occasionally force the extinction of current species and, by creating phenolic acid-like components, impact the survival of nearby species [26]. More precisely, phenolic acids have been shown to induce cell death in the root cells of *Pinellia ternata* by causing the excessive accumulation of ROS [74]. Pyrocatechol and other chemicals have been shown to damage plant photosynthetic systems. They interfere with electron transfer, which in turn reduces the photosynthetic capacity in the plants [43,44]. More research shows that by collaborating with other elements, these phenolics can successfully prevent weed growth even in minute amounts within plants. Phenic acids are essential to plant competition in crops like rice since they function as natural weed control agents [24,75]. Strongly herbicidal phenolic chemicals have been discovered to be present in several plants, such as *Artemisia argyi*. The range of phenolic compounds in *Parthenium hysterophorus* has prompted studies to propose that it may be a useful tool for controlling weeds as well as crops [76]. These discoveries have prompted a great deal of research and advancement in bioherbicide technologies [77].

Researchers studying marine ecosystems mainly believe that phenolic allelochemicals are to blame for the proliferating green tide algae. *U. prolifera* is usually floating in the low UV-B radiation levels in the Yellow Sea region. Protecting algae from UV-B exposure is one of the key functions of phenolic compounds, which enables them to better adapt and survive in changing settings [78]. These algae’s much greater concentration of phenolic compounds will make survival more likely. Phenols are intermediate products of the ascorbate-glutathione (ASA-GSH) cycle, which is how *U. prolifera* activates its antioxidant system to cope with stress, under low-dose and short-term radiation exposure [48,78]. Meantime, *U. prolifera*’s antioxidant system also helps to control photorespiration metabolism and keeps photosynthesis efficient, which allows it to survive and proliferate in unfavorable environments [79]. Part of the reason for the quick adaption and renewal of *U. prolifera* is its genes, which are essential for the manufacture of phenolic chemicals that allow the green macroalgae to spread quickly in their native habitat [80]. On the other hand, eutrophication and acidification of the ocean have proven green macroalgae to be remarkably adaptive [81]. In these situations, algal physiological regulating ability is increased in part by phenolic substances [38]. Furthermore, some research has demonstrated that green macroalgae can successfully regulate the amount of cyanobacteria by producing phenolic chemicals [82]. Phenolic chemicals may influence species community and mutual competition in the whole aquatic ecosystem in addition to the green macroalgae itself [83]. Li et al. (2021) discovered that eugenol significantly inhibited *U. prolifera* spore germination [40]. The ability of phenolic compounds to control the structure of phytoplankton communities and stifle competitors in these conditions emphasizes the critical function of phenolic compounds in preserving ecological balance and having broad ecological consequences [84].

### 2.4. Terpenoids

Through the inhibition of the growth of nearby plants, terpenoids have an impact on biodiversity, community organization, and plant competition [85]. For instance, terpenoids can block the development of root systems and seed germination. It is well recognized that several chemicals, such as betulinic acid, oleanolic acid, and ursolic acid, restrict the growth of several plants, such as *Lactuca sativa*, and *Bidens pilosa*. The main mechanism behind this action is damage to photosystem II in the photosynthetic system of the plant [45]. Significant constituents of volatile organic compounds found in plants and terpenoids also influence the competitiveness, resistance to diseases and pests, and developmental patterns of plants [86]. These allelochemicals are essential to plant communication and defense systems since they can obstruct photosynthetic efficiency, root development, and seed germination in several ways [87,88].

Terpenoids are crucial for the growth, reproduction, and environmental adaptation of *U. prolifera*. As important intermediates in the synthesis of carotenoids, these substances are necessary for green tide algae to survive. Two environmental factors affecting this synthesis system and its downstream pathways, which in turn impact carotenoid synthesis, are light and salt. Liu et al. (2023) have clarified the genetic mechanism of the 2-C-methyl-D-erythritol 4-phosphate (MEP) pathway in green macroalgae, which influences carotenoid synthesis and greatly affects the metabolic control of green tide algae in response to environmental changes [58]. *U. prolifera* is fast-growing partly due to its effective carbon fixation ability, which is intimately related to the terpenoid metabolic pathways [80]. Terpenoids are also required for the strong growth of algae, as research has shown, since they are strongly linked to genes linked to stress in green tide algae [89]. The green tide algae synthesis of terpenes is found to be temperature-dependent [90]. A transcription study showed that genes involved in terpenoid synthesis increased dramatically during spore formation [91]. Specifically, green tide algae create terpenoids that are essential for their physiological processes and may also change the amount of nutrients and dissolved oxygen in the surrounding marine environment [68]. The *U. prolifera* spore germination suppression experiment by Li et al. (2021) also revealed notable inhibitory effects of terpenoids, such as myrcene [40]. Terpenoids have been extensively studied ecologically in algae and microorganisms, even if their precise role in allelopathy among marine species is yet unknown [92].

### 2.5. Other Allelochemicals

**Figure 2:**
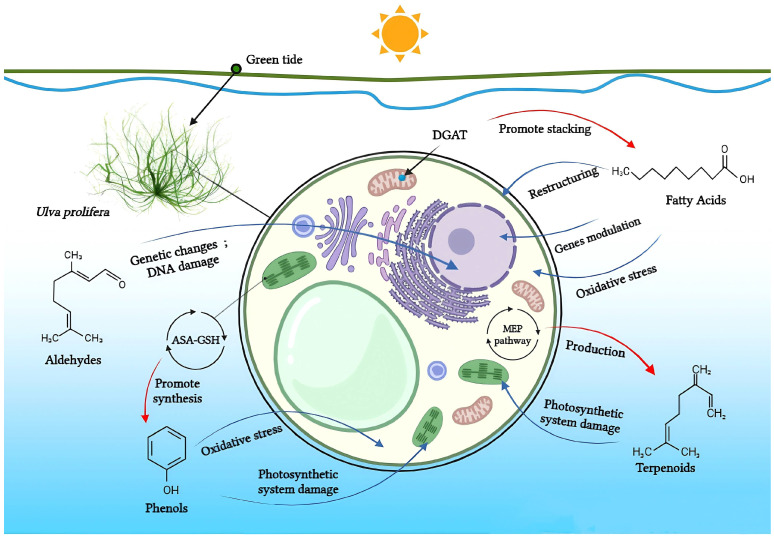
Fatty acids, aldehydes, phenols, and terpenoids in *U. prolifera*: known mechanisms of their allelopathic actions. Allelochemicals’ effect on *U. prolifera*’s physiology is shown by blue arrows; algae’s physiological processes’ effect on allelochemical content is shown by red arrows. (By BioRender).

Halogen compounds, polysaccharides, alkaloids, and other less studied compounds are similarly significant to the ecological balance in the fields of marine ecology and green tides. Although their exact properties are yet unknown, these chemicals may have a big influence on how ecosystems function and how green tides form [68]. Within the green tide alga *U. prolifera*, Figure 2 demonstrates the known mechanisms underlying the allelopathic effects, which exerted by the major four classes of allelochemicals.

Similar to the above-stated substances, polysaccharides interfere with the physiological processes of neighboring plants, therefore preventing them from developing and germinating. These chemicals can be released into the environment by root secretion, volatilization, or plant breakdown, which influences neighboring plants in the soil rhizosphere and changes the interspecific competition pattern [86]. For example, in marine environments, strong allelopathic effects were shown in polysaccharide and protein complexes produced by the marine phytoplankton *H. akashiwo* [93]. While the controlling mechanism is yet unknown, this complex demonstrates how polysaccharides regulate interspecific competition in the aquatic environment by allelopathy. It can attach itself to the cell surfaces of surrounding algae, therefore stifling the growth of rival algae such as *Skeletonema costatum* [93]. Polysaccharides that exhibit allelopathic effects now clearly cling particularly to the target species’ cell surface, drastically restricting their proliferation and altering the number of certain species [94]. Moreover, the marine applications of these polysaccharides have shown considerable potential, such as in aquaculture to strengthen animal immune systems [95]. In-depth research and development of *U. prolifera* polysaccharides can not only help aquaculture thrive and present fresh chances for economic expansion but also provide a remedy for the green tide [96].

Meanwhile, *U. prolifera* can produce dimethylsulfoniopropionate, which is a kind of halogenated substance. The production of these compounds can be achieved by modifying the nutrient solubility in gas and water, together with any potential negative effects on the surrounding biological environment [4]. Sterol concentration similarly rises in response to light stress in *U. prolifera* cells, and this buildup of sterols may likewise cause allelopathic consequences [46]. Octanol was also shown to have some inhibitory impact on spore germination in the experiment of allelochemicals inhibiting *U. prolifera* spore; however, the mechanism of action of these allelochemicals has not been further investigated [40]. In agriculture, plant poisons have been shown by the capacity of some specific alkaloids to suppress seed germination and plant growth. For example, indole alkaloid is one of the substances found in barley that stops weeds and other plants from developing [97]. Similarly, alkaloids such as N-phenylacetamide, cyclo (L-Pro-L-Val), and other diketopiperazine derivatives are present in *U. prolifera* and are believed to be one of the ways to regulate green tides because of their inhibition capability against these green macroalgae [49]. Another alkaloid, Pyrrolopiperazine-2,5-dione, derived from *U. prolifera* has also shown resistance to hazardous red tide microalgae in marine environments [47]. And, allelochemicals from *U. prolifera* including valine, hexanedioic acid, and bis(2-ethylhexyl) ester have strong allelopathic inhibitory effects on *S. alterniflora* growth [33].

## 3. Rapid Detection and Application Prospect of Allelochemicals

Allelochemicals are essential to the process by which green tide algae become the dominating species, even if additional studies are needed to determine their exact mechanism and impacts on the green tide process. A thorough understanding of the mode of action of these chemicals in the natural environment and how they affect the ecological balance of green tide algae and other marine organisms will make new management techniques to effectively control and react to green tide issues and guarantee the ongoing health of marine ecosystems possible. Several sensitive and efficient techniques for chemical detection in water have been established by researchers; these techniques ought to be used for rapid allelochemical in situ detection. Solid phase extraction and ultra-high performance liquid chromatic-tandem mass spectrometry (UPLC-MS/MS) are combined, for example, to enable multiple chemical component monitoring in water with lower detection limits and improved accuracy [98]. Another physical method, including UV absorption-based sensors, can also quickly and accurately identify contaminants in water, such as some phytoallelochemicals [99]. In chemical terms, a less costly and more sensitive assay has been developed by researchers by monitoring glutathione S-transferase activity in aqueous culture systems [100]. Further, the luciferase assay is widely used for field testing since it is very sensitive and easy to use [101]. Using the electrochemical reaction of microorganisms employing microbial fuel cell technology, this biological approach detects harmful compounds in water, including phytoallelochemicals [102]. Currently, existing drone-equipped real-time monitoring systems can effectively collect water samples and quickly perform qualitative and quantitative chemical content analysis [103]. Finally, the use of advanced technologies and algorithms together with a variety of quick probes and automatic alarm systems allows the online biological detection system to provide an instantaneous alarm when anomalies are found, so greatly enhancing the efficacy and precision of water quality monitoring [104,105,106]. These automated, real-time monitoring systems are suitable for routine testing of water quality and also promise to effectively assess and control environmental hazards via the real-time monitoring of changes in plant allelochemicals in reaction to environmental phenomena such as green tides. In the present monitoring and tracking methods for green tide events, two allelochemicals fatty acids and sterol have been utilized as biomarkers to follow *U. prolifera* settlement sites in the later stages of the events [107,108,109].

For green tide monitoring, satellite data applications are the most important methods. These technologies make greater use of satellite resources and considerably reduce operational complexity by utilizing the concepts of open data cubes and real-time data analysis [110]. The VGGUnet model shows how well researchers may utilize deep learning to extract features from data and assess biomass [111]. Furthermore, infrared and synthetic aperture radar imaging techniques enhanced the ability to monitor green tides, such as those employed by the Resources 1-02E and Gaofen-3 satellites, though detecting lower-density algae with these techniques is still difficult [112]. Another disruptive monitoring strategy among the ecological monitoring technologies already being developed is environmental DNA (eDNA) technology. This method utilizes the identification of live tissue DNA release in the surroundings, which may come from a variety of organisms, such as microorganisms and organisms with many cells [113]. And, eDNA technology allowed the researchers to identify certain species in the samples, such as macroalgae [114]. EDNA technology with minimal environmental influence provides a cheap, efficient biodiversity analysis [115,116]. In the investigation of early green tides in the Southern Yellow Sea, Zeng et al. (2023b) successfully tracked changes in *U. prolifera* density and distribution during green tides using eDNA technology [117]. An investigation of these DNA samples made it possible to build an association network encompassing *U. prolifera* and other eukaryotic bacteria [117]. Even though such association studies provide new insights into the interactions between organisms, they now mostly rely on ecological niche theories to explain these interactions. Allelopathic studies in green tides will greatly improve our understanding of these interactions.

**Figure 3:**
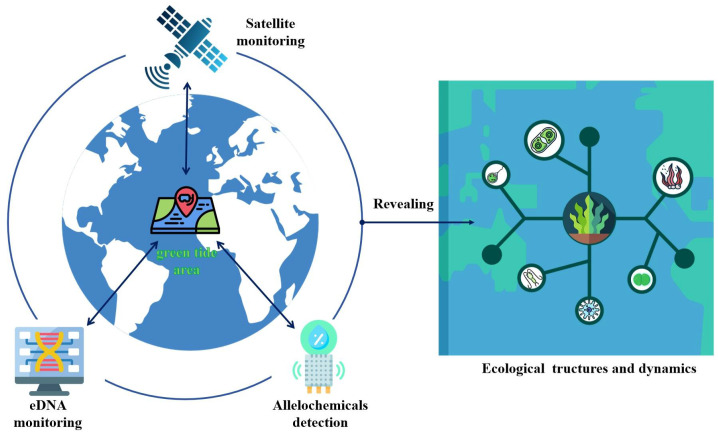
With the confluence of developing technology, ecological adaptation strategies for green tide algae will be better understood. Satellite technology tracks the emergence of green tides regionally; eDNA technology quickly monitors species distribution and community structure; and allelochemical detection greatly fills the gap in the unknown interactions between species. This changes the research emphasis from the traditional nutrient and hydrometeorological factors to the in-depth study of ecological dynamics and structure during green tides.

Whereas satellite technology allows researchers to find and track green tides over a wide area of the sea, eDNA monitoring technology provides an effective way to precisely identify the type and number of organisms in a given area. Particularly crucial during green tides, in situ allelochemical detection can reveal the interactions between algae and other marine life. The integration of satellite technologies and environmental DNA (eDNA) surveillance with rapid allelopathic detection methods represents a significant advancement in ecological monitoring and holds great promise for scientific research and environmental management (Figure 3). Apart from helping scientists to comprehend the growth rates and adaptive strategies of green macroalgae, these data will force a more in-depth analysis of the ecological dynamics and structure and take a front stage compared with traditional nutrient and hydrometeorological factors. All things considered, the coordinated application of these state-of-the-art technologies offers substantial data assistance for studying the ecological processes of green tides.

## 4. Conclusions

Important consequences of allelopathy are seen in the growth of plants and the condition of aquatic environments. Finding out how various substances affect green macroalgae and their surroundings is very important when researching the green tide phenomena. Presently, the most often used allelochemicals in this field are fatty acids, aldehydes, phenols, and terpenes. These compounds have a range of allelopathic effects, from the breakdown of cell membranes to the inhibition of photosynthesis and enzyme activity. However, marine ecology has not given as much attention to allelochemicals such as polysaccharides and alkaloids, which are ubiquitous in terrestrial habitats. Improvement will come from knowing these allelochemicals and their mechanisms better, and our understanding of green tide occurrence and ocean management options will be enhanced, as will the development of new management methods. In addition, with the gradual understanding of allelopathy in green tides, it has become a reality to develop technologies that can detect these allelopathy substances with high accuracy. Combining these high-precision detection techniques with modern environmental monitoring methods, such as eDNA analysis and satellite remote sensing, can not only improve our insight into the dynamics of green tides but also bring new strategies and perspectives for marine ecosystem research. Such integrated monitoring methods can reveal the complex interaction between green tides and their environment, helping us to better understand the biodiversity, ecological function, and ecological balance of marine ecosystems.

## Figures and Tables

**Table 1:** Key allelochemicals in green tide allelopathy.

Allelochemical Classification	Allelochemicals	Chemical Formula	Chemical Structure	Reference
Fatty acid	Palmitic acid	C_16_H_32_O_2_	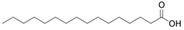	[38,39]
	Oleic acid	C_18_H_34_O_2_	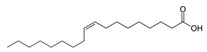	[39]
	Nonanoic acid	C_9_H_18_O_2_	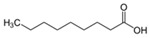	[40]
Aldehyde	Aldehyde 2-trans	/		[41]
	4-trans-decadienal	/		[41]
	Citral	C_10_H_16_O	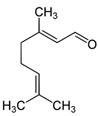	[40]
Phenol	Eugenol	C_10_H_12_O_2_	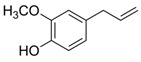	[42]
	Phenol	C_6_H_5_OH	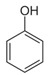	[43]
	Pyrocatechol	C_6_H_4_(OH)_2_	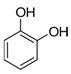	[44]
Terpenoid	Betulinic acid	C_30_H_48_O_3_	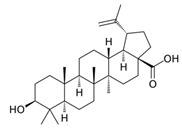	[45]
	Oleanolic acid	C_30_H_48_O_3_	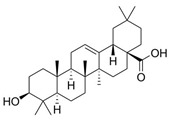	[45]
	Ursolic acid	C_30_H_48_O_3_	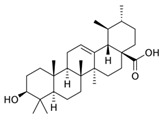	[45]
	Myrcene	C_10_H_16_	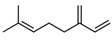	[40]
Alcohol	Sterol	/		[46]
	1-Octanol	C_8_H_18_O	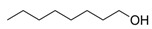	[40]
Alkaloid	Pyrrolopiperazine-2,5-dione	C_7_H_10_N_2_O_2_	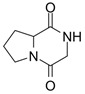	[47]
Amide	N-phenethylacetamide	C_10_H_13_NO	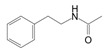	[48,49]
Diketopiperazine derivatives	Cyclo(L-Pro-L-Val)	C_10_H_16_N_2_O_2_	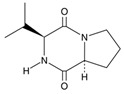	[48,49]
	Cyclo(L-Pro-L-Pro)	C_10_H_14_N_2_O_2_	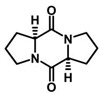	[48,49]
Amino acid	Valine	C_5_H_11_NO_2_	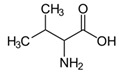	[33]
Carboxylic acid	Hexanedioic acid	C_6_H_10_O_4_	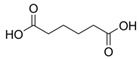	[33]
Esters	Bis(2-ethylhexyl) ester	C_24_H_38_O_4_	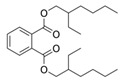	[33]

## Data Availability

Not applicable.
